# Improving the performance of DomainDiscovery of protein domain boundary assignment using inter-domain linker index

**DOI:** 10.1186/1471-2105-7-S5-S6

**Published:** 2006-12-18

**Authors:** Abdur R Sikder, Albert Y Zomaya

**Affiliations:** 1 Advanced Networks Research Group, School of Information Technologies, J12, University of Sydney, NSW 2006, Australia

## Abstract

**Background:**

Knowledge of protein domain boundaries is critical for the characterisation and understanding of protein function. The ability to identify domains without the knowledge of the structure – by using sequence information only – is an essential step in many types of protein analyses. In this present study, we demonstrate that the performance of DomainDiscovery is improved significantly by including the inter-domain linker index value for domain identification from sequence-based information. Improved DomainDiscovery uses a Support Vector Machine (SVM) approach and a unique training dataset built on the principle of consensus among experts in defining domains in protein structure. The SVM was trained using a PSSM (Position Specific Scoring Matrix), secondary structure, solvent accessibility information and inter-domain linker index to detect possible domain boundaries for a target sequence.

**Results:**

Improved DomainDiscovery is compared with other methods by benchmarking against a structurally non-redundant dataset and also CASP5 targets. Improved DomainDiscovery achieves 70% accuracy for domain boundary identification in multi-domains proteins.

**Conclusion:**

Improved DomainDiscovery compares favourably to the performance of other methods and excels in the identification of domain boundaries for multi-domain proteins as a result of introducing support vector machine with benchmark_2 dataset.

## Background

Amino acid composition varies in protein domain region and linker region [1]. Structural domains define the basic building blocks of proteins. Domains are frequently smaller than a protein, yet they epitomize the core principles of the entire protein structure; domains are compact, fold independently and often have a specific function [2-4]. The ability to identify domains is essential, as many types of protein analyses begin with the decomposition of the protein into its functional units – domains. Identification of domains is a complex task and a large number of methods have been developed over last three decades to identify domains from the 3D coordinates of the protein structure [5]. None of the existing structure-based methods performs better than 85% [3]. The task is even more critical and more difficult when the structure of the protein is not known and domains are identified from sequence information alone. A number of sequence-based methods have been developed over the last six years, which identify linker regions between domains; this in turn leads to the identification of domains themselves. Such methods use multiple sources of sequence-based information: for example: DOMpro [6] used evolutionary information (gene-exon shuffling), secondary structure and solvent accessibility information with a recursive neural network; CHOPnet [7] utilizes evolutionary information, amino acid composition and amino acid flexibility analyzed with a neural network; SnapDRAGON [8] predicts domain by using an *ab initio *protein folding method; DomSSEA [9] uses predicted secondary structure; the Nagarajan & Yona [10] method is based on analyzing multiple sequence alignments from database search, position specific physio-chemical properties of amino acids and predicted secondary structure analyzed with a neural network; Galzitskaya & Melnik [11] use side chain entropy of a region to predict domain boundaries; SSEP-Domain [12] predicts domains by combining information of secondary structure element alignments, profile-profile alignments and pattern searches; Armidillo [13] uses amino acid composition to predict domain boundaries; DomCut [1] uses a linker index deduced from a data set of domain/linker segments; finally PRODO [14] uses a neural network from sequence information. Many of the above methods focus exclusively on predicting boundaries for two-domain chains. The overall success rate for sequence-based methods is approximately 25–40% when limited to contiguous domains.

In this present study, we demonstrated that the performance of DomainDiscovery is improved by including inter-domain linker region with a position-specific scoring matrix (PSSM) generated from PSI-BLAST [15], secondary structural information and relative solvent accessibility data. We used secondary structure information and solvent accessibility information based on the assumption that secondary structure elements and level of solvent accessibility in the boundary regions are different from those found in the rest of the protein. The novel features are the use of a SVM (Support Vector Machine) and, most importantly, a unique training set built on the principle of consensus among experts in protein structure.

## Results

The comparison and assessment of domain predictors is complicated by the existence of several domain datasets/databases which sometimes conflict with each other [7]. To test our approach, we performed the following training and testing.

We divided benchmark_2, a dataset containing proteins of known structure for which three methods (CATH [16], SCOP [17] and literature) agree on the assignment of the number of domains, into six clusters of 35 chains where each cluster includes 2-domains, 3-domains, 4-domains, 5-domains and 6-domains chains. One cluster was omitted and the SVM trained using the remaining 5 clusters. This leave-one-out approach was repeated 10 times and the results are averaged. Results are shown on Fig-2 and Fig-3 for DomainDiscovery [unpublished data] and Improved DomainDiscovery respectively. We run each protein chain one by one for all methods. We observed that prediction accuracy increases with window size up to window 19 and then starts to decrease at window size 27. For a window size of 3 the accuracy is 25% for multi domain protein chains, this increases to 65% for a window size of 27 (Fig-2 and Fig-3).

In order to avoid any potential bias in the 6-fold validation test above, a set of 50 chains used for testing (which includes 13 one-domains 20 two-domains, 16 three-domains, 1 four-domain) was assembled by randomly selecting chains from the entire dataset of 315.

In order to compare performance of our method with other methods, we tested the performance of DomainDiscovery and five other methods on a single set of 50 chains (13 1-domain chains; 20 2-doamin chains; 16 3-domain chains; and 1 4-domain chain). The methods were trained on the remaining 265 chains. The performance of each method is presented in Table 3 (in Additional file 1) and summarized in Table 1. To circumvent the issue of non-contiguous domains, which exist in our dataset and which only one of the current methods (SnapDRAGON [8]) addresses, we consider each fragment to represent a separate domain. Thus in the cases of discontinuous domains we artificially increase number of domains predicted by SCOP to match the total number of fragment rather than domains, such cases are labelled with an * in Table 3 (in Additional file 1).

To perform sensitive evaluation of domain boundary prediction methods, we calculate both: presence/absence of domain boundaries and precision of placed boundaries. Our approach Precision of Boundary Placement (PBP) works as follows:

PBP = ((domain boundary in SCOP – domain boundary in the method)/length of the chain) * 100). We introduce NBF (No Boundary Found) if method predicts less number of boundaries or no boundary at all and EBF (Extra Boundary Found) if method predicts more boundary than SCOP domain boundary definition.

We observe that Improved DomainDiscovery performs at 92%, 95%, 88% and 100% accuracy for 1-domain, 2-domain, 3-domain & 4-domain protein chains, respectively and at 92% overall accuracy on the set of 50 chains tested (Fig-4). DomPro [6] performed very well on one-domain protein chains (100% correctly predicted chains) but failed to predict domain boundaries for three or more domain chains (0% of correctly predicted chains). DomPred [9] achieved very good results for one-domain (85%) and two-domain (85%) chains but is less successful in predicting domains boundaries for three-domain chains (25%). CHOP [7] tries to cut chains into more domains than predicted by structural methods. SSEP-Domain [12] performance was superior for one and two domain (85%) chains, but below DomainDiscovery performance for three or more domain chains. Armadillo's [13] tendency is to cut chains excessively as compared to other methods; which makes its performance inferior.

PBP was calculated to determine the average percentage value for the six methods in Table 3 (in Additional file 1). All the percentage values were added for all the chains and divided by the number of domain boundaries predicted by the corresponding method. The precision measurement places Improved DomainDiscovery method in third position relative to other methods (in Table 1), however, the number of NBF and EBF is lower in Improved DomainDiscovery leading to a higher average. The SSEP-Domain method appears to be the most precise in placement of its domain boundaries. However, SSEP-Domain could not find boundaries for a few chains (1a1la, 1a6da, 1avk etc). DomPro performed worst among the six methods. We did not penalize for NBF and EBF however it should be noted that the numbers of NBF and EBF is less in Improved DomainDiscovery than other methods. DomPro [6] has the highest number of NBF (30) and CHOP has the highest number of EBF (38).

An independent test set was evaluated against all methods to avoid the possibility that Improved DomainDiscovery and DomainDiscovery might have an unfair advantage through being trained on part of the test dataset. For independent evaluation we used 21 targets from CASP5. Results for the CASP5 target lists are shown in Table 4 (in Additional file 2), Both DomainDiscovery performed consistently well for one-and two-domains chains. Since all the CASP5 targets have no corresponding domain boundary information in SCOP, we could not perform percentage calculation for some chains, but we can get a grasp from the raw data in the Table 4 (in Additional file 2). We observe that Improved DomainDiscovery performs at 92% and 75% accuracy for 1-domain & 2-domain protein chains, respectively and at 83% overall accuracy on the set of 18 chains tested (Table 2). DomPro[6] (25% for 2-domain and 0% for 3-domain), DomPred (25% for 2-domain and 50% for 3-domain) and SSEP-Domain (50% for 2-domain and 0% for 3-domain) with an accuracy of 67%. Improved DomainDiscovery performed better than existing methods, however, it is noted that the CASP5 targets are predominantly 1-domain protein chains and thus is not an ideal test set for evaluating the prediction of multi-domain protein chains.

## Discussion

While there are many domain boundary assignment methods, none of them are able to delineate domain boundaries for multi-domain protein chains with high reliability. We developed Improved DomainDiscovery to address this issue. A Support Vector Machine (SVM) approach was chosen for its efficiency and consistency. The benchmark_2 dataset [3] which has a high fraction of accurately determined multi-domain proteins provides an excellent training set for our method. Improved DomainDiscovery and DomainDiscovery performed consistently in six-fold validation tests, when compared to other methods, SSEP-Domain [12] performance is superior for single and two-domain chains but inferior for three-domain or larger chains. SSEP-Domain also shows a precise placement of domain boundaries – a subject that will require improvement in our method. SSEP-Domain authors shows that using InterPro [12] pattern searches boosts SSEP-Domain's performance. DomPro [6] predicts domain boundaries accurately for single domain chains. Armadillo predicts single domain chains very poorly but performed reasonably for two-domain chains. In our evaluation another strong method is DomPred [9] which exhibits good performance with single and two-domain chains, but performs poorly with three or more domain chains. Improved DomainDiscovery works better than others because of the efficient use of the powerful machine learning algorithm like SVM and training with clean dataset.

## Conclusion

We have presented an improved protein domain boundary prediction method, DomainDiscovery, based on support vector machine (SVM) and training with structurally defined domains based on consensus among experts. In six-fold cross-validation technique using Benchmark_2 dataset we achieve 70% accuracy for the data that includes single-domain and multi-domain chains. Performance of Improved DomainDiscovery is comparable or better than other recent sequence-based methods, particularly with regards to its performance on multi-domain chains. SSEP-Domain exhibits superior performance but the performance is limited to single and two-domain chains. Improved DomainDiscovery works all the time regardless of the length of the query protein whereas the most of the existing methods can't handle if query protein has a very long chain.

Our future work will focus on improving accuracy of domain boundary prediction by enlarging our training dataset and/or including additional parameters for feature vectors in SVM. Additionally only one of the existing methods SnapDRAGON [8] is capable of predicting discontinuous domains (i.e. domains consisting of more than one fragment) but it is computationally expensive and hence is not suitable for comprehensive sequence analysis. We are working to address assembly of non-contiguous domains from the predicted fragments by post-processing our current results though a probabilistic domain predictor similar to that of DGS[18] (Domain Guess by Size).

## Methods

### Data

Improved DomainDiscovery and DomainDiscovery uses a new comprehensive dataset that was developed for the purpose of benchmarking structure-based domain identification methods[3]. The dataset (referred to here as Benchmark_2) contains proteins of known structure for which three methods (CATH[16], SCOP[17] and literature) agree on the assignment of the number of domains. Benchmark_2 dataset is similar to the dataset published by Holland et.al. [3]. Benchmark_2 comprises 315 polypeptide chains – 106 one-domain chains, 140 two-domains chains, 54 three-domain chains, 8 four-domain chains, 5 five-domain chains and 2 six-domain chains. The dataset is non-redundant in a structural sense: each combination of topologies occurs only once per dataset. Sequences of protein chains are taken from the Protein Data Bank (PDB) [19]. Secondary structure information and solvent accessibility are predicted for each chain in the Benchmark_2 using Sspro [20] and ACCpro [21]. Evolutionary information of each chain is captured in a PSSM (position specific scoring matrix), which was constructed using PSI-BLAST [15]. Inter-domain linker index was taken from DomCut [1]. As an independent assessment of the methods' performance, we analyzed performance of our method and 6 others (DOMPro [6], DomPred [9], CHOP [7], SSEP-Domain [12], Armadillo and a method by Galzitskaya et al. [11]) on 21 targets from CASP5, as described in Table 4 (Additional file 2). Results for Galzitskaya et.al.'s method are taken from their paper [11]. Results for DOMPro [6], DomPred [9], CHOP [7], SSEP-Domain [12] and Armidillo [13] were obtained from their respective web servers.

### Computational approach

#### Support Vector Machine (SVM)

Briefly, SVM is based on the structural risk minimization (SRM) principle from statistical learning theory [22]. It maps the input variable into a high-dimensional feature space using a kernel function and in that space constructs a hyperplane that separates two different classes of feature vectors. In our work, a feature vector represents the position specific scoring matrix, secondary structure information and solvent accessibility values. We used SVM-Light [23] software version 6.01.

**Figure 1:**
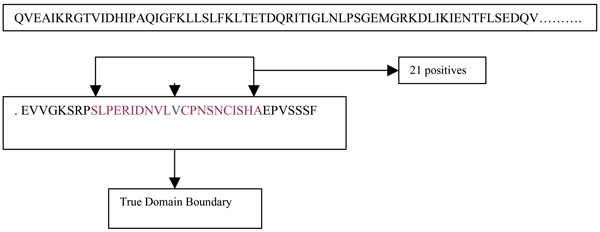
**Example of Input Array for a Window Size of 21**. True boundary here is the residue that SCOP defines as a boundary residue. In case of positive example, we select 10 residues from each side of the true boundary residue and for negative example; we randomly select 21 residues from the rest of the protein chain.

The domain boundary prediction problem can be viewed as a binary classification task, each residue in the protein is labelled either a domain boundary residue or not [6]. The SVM was trained using a window size of 3, 7, 11, 19 and 27 residues, respectively: all residues in the window are considered to be domain boundary residues if the window includes an actual domain boundary (which is defined as a two residue position in Benchmark_2). For each chain, our input is a 1D array I, where the size of I is twice the size of the window. For example, for a window size 21, the size of the input array will be 42 (21 positives and 21 negatives). Fig-1 shows how we select positive and negative examples. Each entry, ***I***_***i***_, is a matrix of dimension 23 encoding the profile (20 values) as well as secondary structure information (helix, strand or coil), solvent accessibility and inter-domain linker index at position *i*.

The following stepwise procedure was employed in the training and testing processes:

(1) Get the protein sequence data.

(2) Run through PSI-BLAST [15], ACCpro [21], Sspro [20] to get PSSM and Solvent Accessibility, Secondary Structure and linker index value form the DomCut [1] paper.

(3) Convert Solvent Accessibility and Secondary Structure information into binary format and add it with PSSM.

(4) Assign labels- positive for boundary residues and negative for non-boundary residues.

(5) Partition the data as training and test set.

(6) Run SVM Learn on training set to create a model.

(7) Run SVM Classify on test set to classify the data using the model.

The following procedure was employed to predict protein domain boundary for a query sequence:

1) Using the model in the above procedure (step 7) we run the classifier module for the query sequence

2) SVM produces individual score for each residue in the sequence

3) If the difference between the two (or more) highest score residues > 0.05 and the location of the two residues is > 30 residues apart then the query protein has more than two domains

4) If the difference between the two highest score residues is > .05 and location of the two residues is < 30 residues apart then the query protein has two domains

5) If the difference between the two highest score residues is < 0.05 then the query protein has single domain

An example of this procedure is shown in Fig-5. Although the example is for two-domain protein, in case of more than two-domain protein the same procedure applies.

### Training, Testing and Validation

**Figure 2:**
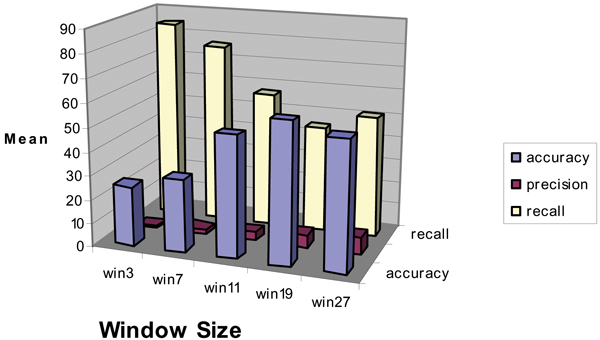
**Six-fold cross-validation results for DomainDiscovery (in average)**. Six-fold cross-validation results for DomainDiscovery; determined by using SVM classifiers for the Benchmark_2 dataset.

**Figure 3:**
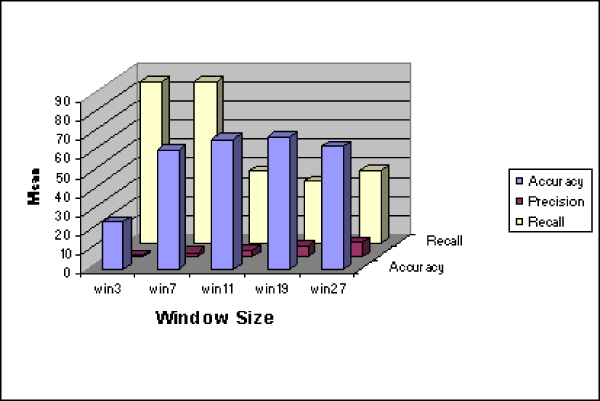
**Six-fold cross-validation results for Improved DomainDiscovery (in average)**. Six-fold cross-validation results for Improved DomainDiscovery; determined by using SVM classifiers for the Benchmark_2 dataset.

**Figure 4:**
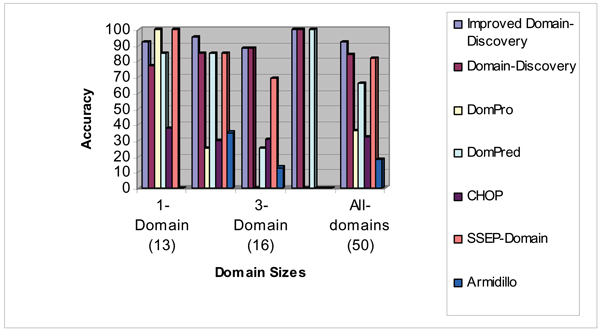
**Percentage of Correct Prediction by different methods**. Percentage of correct predictions for the number of domains defined for each method. If the boundary falls within 30% of the SCOP value the prediction is counted as correct.

**Figure 5:**
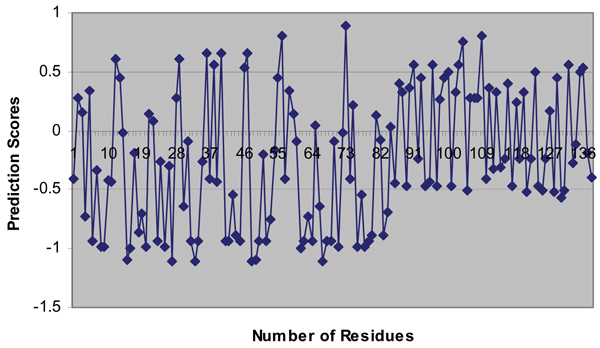
**An Example of Domain Boundary Assignment by Improved DomainDiscovery**. Domain Boundary is at the residue 73.

We use a six-fold cross validation set for training and testing. We divide the Benchmark_2 dataset into six blocks of 35 chains, take one out, train with the remaining five blocks and test the one block that was taken out from the training set. If the predicted domain boundary in the range of ± 15 residues of the true domain boundary then we consider it a correct prediction. We also used a random number generator program to evenly select chains for testing and training. We performed 10 runs training and testing, randomly selecting training and test datasets. For example 17 one-domain 20 two-domains, 9 three-domains, 3 four-domains, 1 five-domain, and 1 six-domains chain for testing and the rest for training including single domain. We tried 5 different window sizes (3, 7, 11, 19 and 27). Results are shown in Fig-3 and Fig-2 for Improved DomainDiscovery and DomainDiscovery methods respectively. We used window size 11 for the testing of the two datasets; results are given in Table 3 (Additional file 1) and Table 4 (Additional file 2).

The performance of the SVM is measured by the accuracy (the proportion of true-positive and true-negative residues with respect to the total positives and negatives residues), the precision (the proportion of the correctly predicted domain boundary residues with respect to the total positively identified residues) and the recall (the proportion of the correctly predicted boundary residues with respect to the total number of domain boundary residues).

Accuracy = (TP+TN)/(TP+FP+TN+FN)

Precision = TP/(TP+FP)

Recall = TP/(TP+FN)

[True-positive (TP), true-negative (TN), false-positive (FP), and false-negative (FN)]

We also tested our methods with 21 CASP5 target lists. Results are shown in Table 4 (in Additional file 2). Summary results for 21 CASP5 targets are shown in Table 2.

## Authors' contributions

ARS developed and implemented the algorithm, prepared datasets, programming in C++, Shell and Perl scripts, interpreted the results, performed testing and drafted the manuscript and AYZ edited the manuscript and introduced the problem initially.

## Supplementary Material

Additional File 1 Comparison performance of domain definition methods for 50 protein chains. Word file. Click here for file

Additional File 2 Results of domain boundary predictions for 21 CASP5 targets. Word file. Click here for file

## Figures and Tables

**Table 1:** Accuracy of boundary placement. Accuracy of boundary placement in average percentages values for chains in Table 3 (in Additional file 1)

Methods	Improved DomainDiscovery Prediction (%)	Domain-Discovery Prediction (%)	DomPro Prediction (%)	DomPred Prediction (%)	CHOP Prediction (%)	SSEP-Domain Prediction (%)	Armidillo Prediction (%)
Accuracy of boundary placement	8.89	13.31	26.58	6.7	9.91	5.47	23.62
Number of NBF	1	1	30	14	7	9	6
Number of EBF	0	2	0	3	38	0	25

**Table 2:** Summary results of 18 chains from CASP5 targets. Summary results of 18 chains from CASP5 targets, average percentage values for chains in Table 4 (in Additional file 2)

No of Domains (Total)	Improved DomainDiscovery Prediction (%)	Domain-Discovery Prediction (%)	DomPro Prediction (%)	DomPred Prediction (%)	CHOP Prediction (%)	SSEP-Domain Prediction (%)	Armidillo Prediction (%)	Galzitskaya et.al. Prediction (%)
1 (12)	92	67	92	83	33	83	0	25
2 (4)	75	75	25	25	0	50	0	25
3 (2)	50	50	0	50	0	0	50	0
All (18)	83	67	67	67	22	67	6	22
