# Supplementary material for: Improving the performance of DomainDiscovery of protein domain boundary assignment using inter-domain linker index
Source: BMC Bioinformatics. 2006 Dec 18;7(Suppl 5):S6. doi: 10.1186/1471-2105-7-S5-S6 (PMC1764483; doi:10.1186/1471-2105-7-S5-S6)
Supplement: Additional File 2 — Results of domain boundary predictions for 21 CASP5 targets. Word file. [file 1471-2105-7-S5-S6-S2.doc]

**Table 4. Results of domain boundary predictions for 21 CASP5 targets.** Pdbid is the Protein Data Bank identification code followed by the chain identifier. In the fourth column the number in bold is the number of domain followed by domain boundary definitions according to SCOP. An * indicates that the number of domains includes fragments associated with non-contiguous domains for instance, 3* means two SCOP domains where one domain has a fragment. In columns four to nine, NBF means No Boundary Found and EBF means Extra Boundary Found. Percentage value represents the difference between the reference and assigned domain boundary: PBP (Precision of Boundary Placement) = ((domain boundary in SCOP – domain boundary in the method) / length of the chain) * 100. Values above 30% are counted as incorrect predictions.

| **No** | **pdbid** | **Target** | **SCOP** | **Improved DomainDiscovery** | **Domain-**  **Discovery** | **DOMpro** | **DomPred** | **CHOP** | **SSEP-Domain** | **Armidillo** | **Galzitskaya et.al.** |
| --- | --- | --- | --- | --- | --- | --- | --- | --- | --- | --- | --- |
| 1 | 1izm | T0129 | **1**  182 | **1** (1%)  181 | **2** (55%,EBF)  81 | **1** (0%)  182 | **1** (0%) | **1** (1%)  182 | **1** (1%)  181 | **2** (73%,EBF)  50 | (39%)  111 |
| 2 |  | T0134 | 259 | **1** | 46 | 86 | **1** | **1**  249 | **1** 250 | 82 | 92 |
| 3 |  | T0145 | **1**  187 | **1** (0%)  187 | **2** (66%,EBF)  63 | 70 (68%,EBF) | **1** | NBF | **2** (39%)  114 | 40 (79%) | 130 (30%) |
| 4 | 1nrk | T0146 | 325 | **1** (0%)  325 | **3**(EBF, 15%)  149,277 | **1**  325 | **1** | **1**  308 | **1**  324 | 200 | 200 |
| 5 | 1m65 | T0147 | **1**  245 | **1** (0%)  245 | **1** (0%)  245 | **1** (0%)  245 | **2** (EBF,68%)  79 | **2** (EBF,22%)  190 | **1** (0%)  244 | **4** (EBF,EBF,  46%)  48,100,132 | (EBF,53%)  115 |
| 6 | 1ino | T0148 | **2**  89 | **2** (0%)  82 | **2** (15%,)  52 | **2** ( 65%)  62 | **1** | **1** (0%)  162 | **2**(7%)  162 | **2** (EBF, 54%)  80 | (EBF, 60%)  70 |
| 7 | 1nij | T0149 | **2**  223 | **2** (13%)  181 | **2** (20%)  287 | **1** (NBF,0%)  318 | **2** (4%)  211 | **3** (EBF,0%)  184,223 | **2** (3%)  213 | **2** (39%)  100 | (34%)  115 |
| 8 |  | T0158 | 319 | **2**  66 | **2** 84 | **1** 319 | **2**  198 | **3**  83,275 | **1** 318 | **2** 84 | 73 |
| 9 | 1r9q | T0159 | **1**  309 | **1** (0%)  309 | **1** (0%)  309 | **1** (0%)  309 | **1** | **1** (2%)  302 | **1 (**0%)  308 | **3** (EBF, 29%)  135,220 | (25%)  232 |
| 10 | 1mw5 | T0161 | **1**  156 | **1** (0%)  156 | **2** (EBF,33%)  122 | **1** (15%)  156 | **1** | **1**  156 | **2** (EBF,16%)  155 | **2** (EBF, 37%)  115* | (EBF, 59%)  74 |
| 11 | 1izn | T0162 | **1**  286 | **1** (0%)  286 | **1** (0%)  286 | **1** (0%)  286 | **1** | **4**  85,138,217 | **1** (0%)  285 | **4** (EBF,EBF,EBF,35%)  76,110,140,185 | (29%)  202 |
| 12 | 1l7a | T0165 | **1**  318 | **1** (0%)  318 | **1** (0%)  317 | **1** (0%)  318 | **2**(EBF,67%)  105 | **2** (EBF,87%  42 | **1** (0%)  317 | **3** (EBF,76%)  45,75 | (38%)  197 |
| 13 | 1m33 | T0171 | **1**  258 | **1** (0%)  258 | **1** (0%)  258 | **1** (0%)  256 | **1** | **1**  230 | **1** (0%)  255 | **2** (EBF,55%)  116 | (EBF, 59%)  107 |
| 14 | 1m6y | T0172 | **3**  114,216 | **3** (0%,6%)  114,235 | **2**(NBF, 4%)  203 | **2** (NBF, 11%)  250 | **3** (2%,9%)  107,189 | **1** (NBF,25%)  291 | **1** (NBF, 27%)  298 | **3** (25%,6%)  40,235 | (3%,NBF)  105 |
| 15 | 1q74 | T0173 | **1**  303 | **2** (EBF, 54%)  138 | **2**(EBF, 72%)  84 | **1** (0%)  303 | **1** | **2** (EBF,40%)  182 | **1** (0%)  302 | **2** (EBF, 44%)  170 | (EBF, 48%)  158 |
| 16 | 1mg7 | T0174 | **2**  187 | **2** (0%)  187 | **2** (16%)  254 | **2** (32%)  320 | **1** | **3** (0%,EBF)  186,372 | **3** (5%,EBF)  165,220 | **4** (8%,EBF,EBF)  220,250,350 | (12%)  235 |
| 17 | 1mw7 | T0177 | **1**  240 | **2** (EBF, 63%)  88 | **1** (15%)  205 | **1** (0%)  240 | **1** | **3** (EBF,51%)  78,118 | **1** (0%)  239 | **3** (EBF,50%)  40,120 | (55%,EBF)  107 |
| 18 | 1iy9 | T0179 | **1**  276 | **1** (0%)  276 | **1** (13%)  240 | **1** (13%)  240 | **1** | **3** (EBF,15%)  90,235 | **1** (0%)  275 | **3** (EBF,42%)  70,160 | (EBF,69%)  86 |
| 19 | 1o12 | T0186 | **3** 43,331 | **2** (NBF,62%)  105 | **3** (1%, 36%)  48,199 | **1** (0%)  364 | **2** (NBF,39%)  188 | **NBF** | **1** (0%)  363 | **2** ( 1%,NBF)  48 | (NBF,18%)  267 |
| 20 | 1o0u | T0187 | **2**  249 | **1** (NBF, 40%)  417 | **1** (0%,NBF)  417 | **2** (30%)  289 | **3** (EBF,53%)  75,198 | **3** (EBF,40%)  39,249 | **1** (0%)  416 | **3** (EBF, 14%)  200,355 | (EBF,16%)  349 |
| 21 |  | T0194 | **1**  237 | **1** (0%)  237 | **1**  236 | **1**  237 | **1** | **2** (EBF)  237 | **1**  236 | **2** (EBF,49%)  120 | (47%,EBF)  125 |
